# Hormonal transitions across the lifespan shape susceptibility to Sjogren’s disease

**DOI:** 10.1093/rheumatology/keag087

**Published:** 2026-02-18

**Authors:** Eliza C Diggins, Melodie L Weller

**Affiliations:** School of Dentistry, University of Utah, Salt Lake City, UT, USA; School of Dentistry, University of Utah, Salt Lake City, UT, USA; Division of Microbiology and Immunology, Department of Pathology, University of Utah, Salt Lake City, UT, USA

**Keywords:** Sjogren’s disease, Sjogren’s syndrome, sex hormones, hormone-mediated pathogenesis, autoimmunity, sex hormones, testosterone, oestrogen, sex bias, hormonal regulation

## Abstract

**Objectives:**

Sjogren’s disease (SjD) shows a strong female predominance, but the contribution of age-related hormone changes to this sex bias remains uncertain. We investigated whether natural hormonal transitions across the lifespan align with variation in male and female prevalence of SjD.

**Methods:**

Electronic health records from 101 856 SjD patients and 1.33 million controls were analysed. Sex-specific prevalence was compared with serum testosterone, oestradiol and SHBG levels. Population-level hormone distributions from the National Health and Nutrition Examination Survey (NHANES) were incorporated using imputation. Generalized linear models evaluated associations between hormone fluctuations and sex prevalence across age groups.

**Results:**

Male prevalence among SjD patients peaked during early childhood (30.1% [95% CI: 26.2–34.1]), declining sharply in late puberty into adulthood (9.8% [95% CI: 9.5–10.2]) and rose again in older adults (13.5% [95% CI: 13.3–13.8]). These non-linear shifts paralleled age-dependent trajectories of testosterone and oestradiol. Hormone concentrations did not differ significantly between SjD patients and controls, indicating that physiological transitions, rather than abnormal levels, align with disease risk.

**Conclusion:**

Age-dependent hormonal changes correspond with evolving sex bias in SjD, challenging the static 9:1 female-to-male paradigm. These findings highlight the role of age-related hormonal dynamics in shaping autoimmune susceptibility.

## Graphical abstract

**Graphical Abstract:**
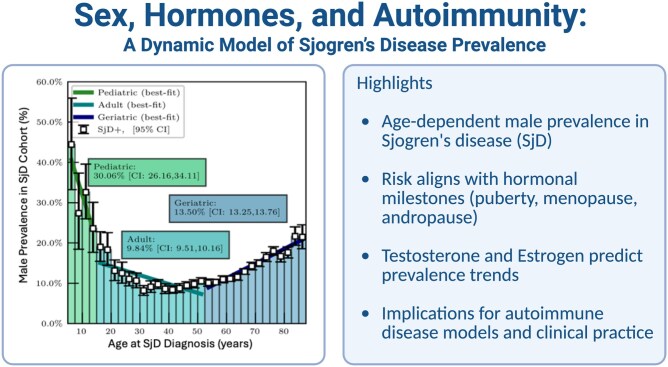

Rheumatology key messages

- In Sjogren’s disease (SjD), female prevalence rises after puberty and peaks around peri- and post-menopause.
- SjD sex ratios track hormonal transitions, implicating testosterone and oestradiol in susceptibility.
- In SjD, males reach 30% in childhood and represent an increasing proportion after age 52.5.

## Introduction

Sjogren’s disease (SjD) is a complex autoimmune syndrome characterized by immune-mediated damage to mucosal tissue and moisture-secreting glands, resulting in dry eyes and mouth, reduced tear production and various glandular and extra-glandular symptoms [1]. The underlying aetiology of SjD is multifactorial, and often attributed to a combination of genetics, hormone profiles and chronic pathogen exposure [2, 3]. SjD also exhibits a strong female sex bias, with ≥85% of patients being female, and most often diagnosed during peri- or postmenopausal timepoints, raising the hypothesis of a sex hormone-mediated mechanism contributing in part to disease susceptibility [4, 5]. However, the mechanisms underlying this sex bias remain unclear.

Previous studies have reported mixed findings regarding sex hormone abnormalities in SjD patients compared with the controls, posing challenges in defining sex hormone-driven aetiological factors [6–9]. Given the variability across studies and the lack of consistent, measurable sex hormone abnormalities associated with SjD, we pursued a different approach. We hypothesize that age-related sex hormonal fluctuations (oestrogen and testosterone), such as those occurring during puberty, menopause and andropause, modulate susceptibility to develop SjD, rather than persistent or generalized sex hormone abnormalities across the patient population.

To test this hypothesis, we analysed over 100 000 SjD electronic health records (EHRs) and over 1.3 million non-SjD controls from the TriNetX Research Network (TNXRN), combined with National Health and Nutrition Examination Survey (NHANES) baseline hormone data. Our findings reveal striking age-dependent shifts in SjD sex bias, particularly in paediatric and older populations, emphasizing the need for further research into hormone-associated immune modulation in SjD pathogenesis.

## Methods

We analysed sex-specific prevalence and hormone associations in SjD using patient records from the TNXRN and baseline hormone data from NHANES. Details on complete cohort selection, data processing and statistical methodologies, including male sex prevalence calculation and hormone imputation, are provided in Supplementary Data S1. The study was conducted with ethical approval from the Institutional Review Board (IRB_00170807) at the University of Utah. This is a retrospective study and consent was not obtained as only de-identified data was utilized in analyses.

Briefly, the SjD cohort (*N* = 101 856) included patients with at least two M35.0* ICD-10-CM codes (or subcodes) spaced by at least 6 months. Non-SjD controls were selected from patients presenting with SjD-associated sicca symptoms (dry eye, dry mouth or keratoconjunctivitis) but without a formal SjD diagnosis. This strategy was used to minimize confounding factors related to symptom-driven healthcare utilization and diagnostic testing rates, ensuring that both the groups were comparable in terms of clinical engagement and testing likelihood. This approach provided a more rigorous comparator than a general healthy population, although it may bias towards underestimating true differences between the groups.

## Results

We analysed electronic health records from 101 856 patients diagnosed with SjD and over 1.3 million non-SjD controls identified from the TNXRN dataset (Table 1). The SjD cohort was predominantly female (87.56%) compared with 63.57% female in the control cohort (*P* < 0.0001). The average age of SjD patients was significantly higher at 58.40 years (±15.12 S.D.) compared with 54.94 years (±21.16 S.D.) for the non-SjD controls (*P* < 0.0001). Racial demographics indicated that the majority of SjD patients identified as White (63.42%), followed by Asian (13.08%) and Black or African American (8.25%), with significantly different distributions compared with the controls (*P* < 0.0001). Ethnicity data revealed a lower proportion of Hispanic or Latino patients in the SjD group (5.88%) compared with the control group (10.46%; *P* < 0.0001).

**Table 1:** Demographic comparison of SjD and non-SjD control cohort.

	SjD (%)	Non-SjD controls (%)	
	*N* = 101 856	*N* = 1 333 082	*P*-value
Sex^a^			<0.0001
Male	12 667 (12.44)	485 608 (36.43)	
Female	89 189 (87.56)	847 474 (63.57)	
Race^a^			<0.0001
Amer. Indian/Alaska Native	410 (0.40)	5271 (0.40)	
Asian	13 319 (13.08)	61 823 (4.64)	
Black or African Amer.	8404 (8.25)	184 959 (13.87)	
Native Hawaiian or P.I.	134 (0.13)	2936 (0.22)	
White	64 601 (63.42)	834 667 (62.61)	
Unknown	14 988 (14.71)	243 426 (18.26)	
Ethnicity^a^			<0.0001
Hispanic or Latino	5993 (5.88)	139 403 (10.46)	
Not Hispanic or Latino	75 564 (74.19)	898 946 (67.43)	
Unknown	20 299 (19.93)	294 733 (22.11)	
Age,^b^ *µ* [S.D.]	58.40 [15.12]	54.94 [21.16]	<0.0001
Age groups^a^			<0.0001
0–10	537 (0.53)	89 460 (6.71)	
11–20	2065 (2.03)	60 229 (4.52)	
21–30	5830 (5.72)	93 099 (6.98)	
31–40	10 855 (10.66)	120 574 (9.04)	
41–50	18 073 (17.74)	179 310 (13.45)	
51–60	25 981 (25.51)	270 557 (20.30)	
61–70	25 539 (25.07)	309 916 (23.25)	
71+	12 976 (12.74)	209 937 (15.75)	

a Pearson’s χ^2^ tests were performed on each demographic feature (sex, race, etc.) as well as on each of the labels of each feature (male, female, etc.).

b Kolmogorov–Smirnov test.

Among the SjD cohort, 540 paediatric patients (ages 0–15 years) were identified, with hormone data (oestrogen, testosterone, SHBG) available for 33 individuals. In contrast, the next age group (15–52.5 years) included over 3700 SjD patients with available hormone data. Although overall hormone testing rates were low across all the age groups, rates of sex hormone testing were similar across the age and sex groups, indicating no major testing bias over time, enabling a comparative analysis.

**Figure 1:**
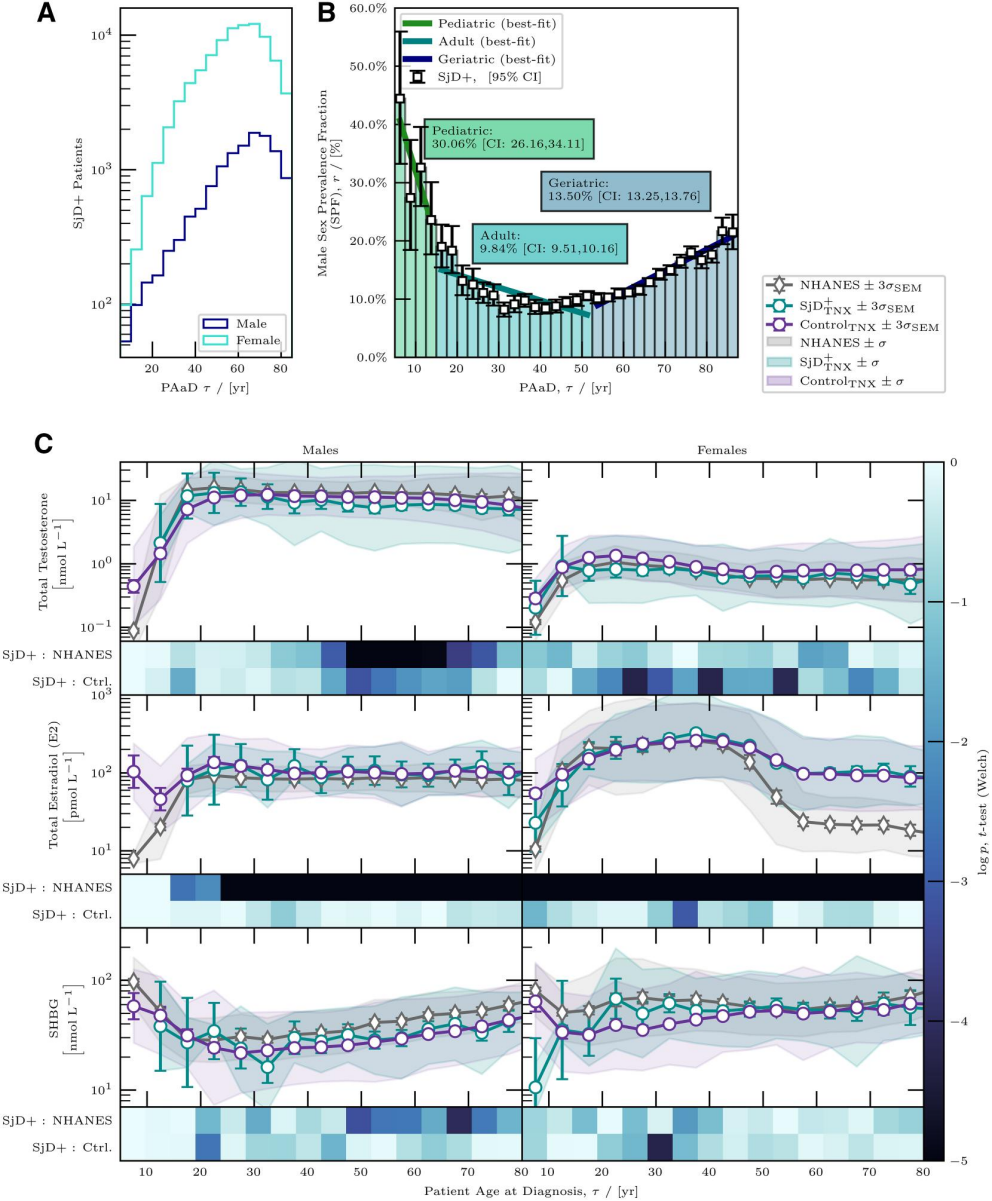
Age-based sex bias and serum hormone levels in SjD. (**A**) Age-based male sex prevalence (SPF) in SjD and (**B**) timeline of SjD diagnosis with 95% credible intervals, shown in 2.5-year age bins from ages 5 to 90. Categories include paediatric (<15 years), adult (15–52.5 years) and geriatric (>52.5 years). Best-fit OLS lines for each group indicate trends, with slopes of −2.29%, −0.22%, and 0.36% per year, respectively, calculated using Bayesian methods. (**C**) Mean serum hormone levels (SHLs) for testosterone, oestradiol and SHBG) cross SjD, control and NHANES cohorts, displayed by sex. Heatmaps show *P*-values (Welch’s *t*-test) for differences across bins, with SHLs modelled as log-normal and MLE applied for means. Error bars represent 3σ confidence intervals, and shaded areas indicate 1σ standard deviations. Similar SHLs between the SjD+ and control groups, diverging from NHANES, suggest testing bias

Analysis of the age-dependence in male SjD prevalence indicated a dynamic interaction between sex hormone levels and disease susceptibility across the lifespan (Fig. 1). Male prevalence peaks in paediatric patients (ages 5–15 years) at 30.1% [95% CI: 26.2–34.1], declines to its lowest in adults (ages 15–52.5) at 9.8% [95% CI: 9.5–10.2] and rises again in older patients (ages 52.5+ years) to 13.5% [95% CI: 13.3–13.8] (Fig. 1A and B). Most paediatric male SjD diagnoses occurred under age 10. Hormone measurements (oestradiol and SHBG) were sparse for this subgroup, limiting interpretation of early childhood hormone patterns. These trends indicate that male prevalence varies across the lifespan, with a decrease of −2.3% per year during paediatric ages and a gradual increase of 0.36% per year in older adults. The sex prevalence fraction (SPF) curve closely mirrors age-related fluctuations in serum testosterone and oestradiol levels, emphasizing that hormone transitions coincide with age-related shifts in sex prevalence.

Serum hormone levels (SHLs) were consistent between the SjD and control groups. Fig. 1C shows SHLs for testosterone, oestradiol and SHBG in the SjD, non-SjD control and NHANES cohorts. A slight reduction in testosterone was noted in SjD males aged 40–60 compared with the controls, though this effect was marginal (Cohen’s *d* ≈ 0.5) and not significant. SHBG and oestradiol levels also showed minimal variation from the controls, with overlapping confidence intervals across the age groups. Differences were observed between both the male and female cohorts and the NHANES cohort, suggesting evidence of a testing bias. These results suggest that deviations in testosterone, oestradiol or SHBG are unlikely to be primary factors in SjD development. Instead, the observed prevalence patterns more likely reflect an association with normal sex hormone fluctuations, age-related immune response changes and other mechanisms underlying disease pathogenesis.

Best-fit hormonal models for the SPF confirmed a strong correlation between SPF and SHLs of both oestradiol and testosterone (Supplementary Fig. S1). Goodness-of-fit metrics were comparable between the two hormones, with oestradiol showing a marginally better fit (AIC: 21.43 for oestradiol vs 21.69 for testosterone; BIC: −111.37 for oestradiol vs −111.11 for testosterone), suggesting that fluctuations in oestradiol may play a slightly more prominent role in modulating susceptibility. Additionally, the residuals were normally distributed for both models, indicating that they were unbiased and accurately captured the trends in the data. These results further support the hypothesis that typical age-related fluctuations in sex hormones may contribute to the modulation of sex-specific SjD prevalence across different age groups.

## Discussion

This study identifies a striking shift in the prevalence of SjD among males, with paediatric SjD patients exhibiting a male prevalence of 30.06%, which sharply declines to 9.85% in adults. This dramatic shift highlights the potential role of age-related hormonal changes, particularly androgens, in modulating disease susceptibility. Prior studies have noted a higher male prevalence in paediatric SjD, but with smaller sample sizes and a less pronounced sex disparity. For instance, Ramos-Casals *et al.* characterized a paediatric SjD cohort of 158 children, reporting 13.9% male prevalence [10], while Basiaga *et al.* analysed 300 paediatric SjD cases, identifying 17% male prevalence [11]. Additionally, Means *et al.* systematically reviewed paediatric SjD cases, with individual studies reporting male prevalence ranging from 15% to 25% [12]. Our findings, based on a significantly larger cohort, provide stronger epidemiological evidence for this sex-based shift and further support the hypothesis that hormonal regulation may play a key role in SjD pathogenesis.

Our findings highlight a strong association between age-related hormonal modulation and sex-specific SjD prevalence. Rather than viewing the sex bias in SjD as static, our data suggest an evolving interplay between testosterone, oestradiol and SHBG levels across different life stages, modulating disease susceptibility. This perspective challenges traditional models that consider sex bias as a fixed prevalence with a 9:1 female-to-male ratio and instead frames it as age-dependent, associated with natural hormonal fluctuations rather than deviations from expected levels.

The findings from this study are consistent with the hypothesis that testosterone and other androgens may influence susceptibility to SjD. During puberty, when testosterone levels surge in males, the parallel decrease in SjD prevalence suggests a potential protective effect of androgens [13]. Conversely, as testosterone levels decline with age, the gradual increase in male SjD prevalence aligns with a role for androgens in immune modulation [14]. Further supporting this hypothesis, males with Klinefelter syndrome (47,XXY), who have inherently lower androgen levels and increased X-chromosome gene dosage, exhibit autoimmune profiles more similar to females, including increased susceptibility to SjD [15, 16]. Notably, case reports suggest improvement with testosterone therapy, although these findings are limited and not definitive [17–20]. This may be due to challenges in achieving the correct balance of testosterone, oestrogen and free SHBG and ability to target delivery to disease affected tissues rather than a lack of efficacy. Additionally, other factors such as chronic inflammation, epigenetic changes and autoantibody-mediated tissue damage may contribute to immune dysregulation beyond hormonal effects.

The testosterone-to-oestrogen ratio may be an important factor in modulating SjD susceptibility, particularly in women during menopause, when sex hormone levels undergo a drastic shift. In adult women, testosterone declines steadily, while oestrogen levels remain relatively stable until menopause, when they sharply decrease [21]. This sudden oestrogen drop alters the testosterone-to-oestrogen ratio, increasing relative androgenic influence. However, this shift may not be beneficial if free testosterone is concurrently reduced by increasing SHBG levels, as testosterone binds to SHBG with higher affinity compared with oestradiol. SHBG follows a U-shaped concentration curve across the lifespan, declining from early adulthood to midlife but rising again in later age [22]. Because oestrogen stimulates SHBG production, the sharp decline in oestrogen during menopause initially reduces SHBG levels. However, as ageing progresses, SHBG levels increase [22], further binding free testosterone and reducing its bioavailability. If testosterone contributes to protection against SjD, then the combined effect of increasing SHBG and declining oestrogen may lead to reduced bioavailable testosterone during menopause, which could help explain the higher prevalence of SjD in postmenopausal women. These findings raise the possibility that interventions targeting testosterone-to-oestrogen ratios or SHBG-mediated reductions in free androgens could be explored as potential strategies for SjD prevention or management, though further studies are needed.

SjD likely results from a combination of genetic susceptibility, hormonal regulation and environmental exposures [2, 3]. Although this study identified associations between age-dependent sex hormone profiles and SjD prevalence, hormones alone are insufficient to trigger disease onset. Instead, sex hormone fluctuations may modulate immune or epithelial responses in individuals with underlying genetic or environmental risk factors, contributing to disease susceptibility.

Limitations of this study include reliance on provider-based diagnoses, which may vary across healthcare settings, potentially affecting diagnostic accuracy. Lower SjD prevalence observed among Hispanic and Black patients may reflect differences in healthcare access, diagnostic bias or clinical presentation. Our use of symptomatic, non-SjD controls was designed to match healthcare-seeking behaviour and symptom profiles between the groups. This approach may have included individuals with early, undiagnosed or atypical SjD, potentially reducing detectable differences between cohorts and emphasizing the need for caution in interpreting small or non-significant hormonal differences. While our imputation approach corrected for potential selection bias due to low testing rates, it inherently relies on the assumption that our patient cohorts closely match the NHANES general population in sex hormone profiles. This could potentially deviate from clinically tested hormonal differences between SjD patients and the controls. Furthermore, systemic hormone measurements may not fully capture local tissue-specific hormone activity within exocrine glands relevant to SjD pathogenesis.

These findings have important clinical implications, suggesting that age-related hormonal transitions could be leveraged to improve early identification and personalized management of SjD. Our findings suggest that natural hormonal transitions, particularly during prepubescence, menopause and andropause, may represent windows of heightened susceptibility to SjD. Monitoring hormone levels during these periods could potentially identify individuals at greater risk for disease development or progression. Although clinical interventions based on hormone modulation remain speculative, our results highlight the potential for sex hormone-focused strategies to complement existing diagnostic and therapeutic approaches. Future studies should evaluate whether interventions targeting androgens could offer protective effects, especially in paediatric male and postmenopausal female populations. Incorporating hormone-aware risk assessments into SjD management strategies may ultimately facilitate earlier detection and more personalized therapeutic approaches.

## Supplementary Material

keag087_Supplementary_Data

## Data Availability

This study used de-identified electronic health record data from the TriNetX Research Network, which are available only to institutions with licenced access and cannot be shared publicly by the authors. NHANES hormone datasets used for baseline estimates are publicly accessible through the Centers for Disease Control and Prevention at https://wwwn.cdc.gov/nchs/nhanes/.
